# Are you ready for a cyberattack?

**DOI:** 10.1002/acm2.13422

**Published:** 2021-09-10

**Authors:** Daniel W. Pinkham, Ina M. Sala, Emilie T. Soisson, Brian Wang, Matthew A. Deeley

**Affiliations:** ^1^ Department of Therapeutic Radiology Yale University School of Medicine New Haven Connecticut USA; ^2^ Department of Radiation Oncology University of Vermont Medical Center Burlington Vermont USA

We are undoubtedly all familiar with the concept of computer “viruses,” “malware,” and “phishing,” and possibly even “ransomware.” They may have affected us in small or large ways personally, but many of us likely have neither truly considered nor prepared for the disaster scenario that a cyberattack could present to our patients and departments. These attacks are no longer a theoretical risk but rather a present reality^1^, ^2^ for which we must prepare now. The aim of this editorial is to share the experiences of three institutions facing cyberattacks in the last year in the hope these case studies may help frame the conversation at your own institutions.

## 1 INSTITUTION A

This radiation oncology department has five regional sites and the main campus, treating approximately 260 patients per day on 9 Varian and 3 Elekta Linacs. Mosaiq Cloud (Elekta, AB., Stockholm, Sweden) is utilized as the record and verify (R&V) system and Eclipse Cloud (Varian, Inc., Palo Alto, CA) as the treatment planning system (TPS). In late April 2021, this institution halted patient treatments for all six sites because the Mosaiq cloud was compromised by a cyberattack targeting its datacenters. The hospital network, treatment planning platforms, and linear accelerators on site were not compromised by this attack, but the sudden disruption of the oncology information system (OIS) across the hospital network caused an immediate cessation of all treatments while the radiation oncology team evaluated its recovery plan.

### 1.1 Recovery strategies

Patients on the Elekta Linacs were transferred to a nearby independent hospital with a beam‐matched machine. This Elekta transfer process began 2 days after the initial disruption and was spread out over 3–4 clinic days to allow sufficient time for data migration, recalculation, and plan checks. The first patient's plans on the Varian TrueBeam were transferred from Eclipse using a clinical file mode, which also began 2 days after the disruption. This allowed the patients’ treatment plans to be transferred to the linacs in their entirety, but R&V capabilities were no longer available. Therefore, a comprehensive checklist and paper chart were created prior to treatment for each patient. All secondary checks, patient‐specific Quality Assurance (QA), and chart checks were performed again. Early attempts at restoring the original Mosaiq cloud were unsuccessful, and 5 days after the disruption when the vendor promised to restore it, the vendor informed this institution that the restoring would take at least one more week. Then, this institution decided to rebuild a new local Mosaiq server from scratch.

Thirteen days after the initial disruption, after significant OIS commissioning effort, all Elekta Linacs were ready to resume treatments on the new local server, while Varian Linacs continued treatments in file mode. By day 20, all Varian Linacs were commissioned on the new server. Patients whose plans had been migrated into file mode continued to receive treatments that way until termination of the full treatment, or unless a cone‐down plan was starting. At the same time, all new patient treatment courses were initiated on the new local Mosaiq server. The decision to operate two R&V methods concurrently was not made lightly: the inherent risk of this additional complexity was evaluated against the time and resources needed to manually repopulate all ongoing treatment records into the new server when the entire staff has experienced extended fatigue. The last fraction delivered in file mode throughout all six sites was 57 days after the initial interruption, and normal clinical operation resumed.

### 1.2 Patient impact

Most patients had their treatments delayed by at least 2 days, with some delays exceeding 2 weeks after all plans could be successfully migrated, replanned, and checked. Additionally, patients who were transferred to other sites had to travel considerably longer distances than usual. Lastly, team fatigue was of significant concern, with nearly all team members contributing extended hours for a prolonged time period.

Several resources were used to track the cumulative dose before the interruption, such as (1) weekly physician on‐treatment visit (OTV) notes that were transferred to the hospital's electronic medical record software, (2) miscellaneous patient notes and records (physical and electronic) kept by therapists and physicians, and (3) an internal Excel planning spreadsheet maintained by dosimetrists. Later, Elekta provided the institution a treatment summary spreadsheet extracted from the original Mosaiq cloud system. This provided a comprehensive database of patients on treatment throughout the hospital network, including prescribed doses/fractions, delivered doses/fractions, treatment site information, and treatment start dates. The final update for this database had occurred within 3 min of the nominal cyberattack time identified by Elekta.

## 2 INSTITUTION B

This radiation oncology department houses one Elekta linear accelerator with one Siemens CT simulator, is staffed by approximately 12 employees, and treats roughly 20 patients per day. They utilize the Varian Eclipse TPS and the Elekta Mosaiq R&V, both installed locally.

In late March 2021, the clinical team was notified that the IT department had shut down hospital servers after malicious software activity was caught propagating throughout the entire hospital network earlier in the morning. Approximately 10 office computers were compromised in the radiation oncology department alone. On some, files were unreadable or were fully encrypted by the cyberattack. Hospital servers used for radiation oncology department file sharing, treatment planning, machine QA, and patient‐specific QA were shut down by the IT department until additional security software could be installed. It was later determined that the Eclipse server was fully compromised and data backup/restoration efforts would have to be pursued. The extent to which the Mosaiq server was compromised was not fully apparent to the clinical team, but the IT department notified them that a recent pristine backup was readily available and they would restore that shortly.

Treatment delivery systems (LINAC, IGRT, and C‐RAD) fortunately were unaffected, either because they were secured behind a vendor firewall, were powered off, or because they did not reside directly on the hospital's IT domain. The department's CT simulator was also powered off during the cyberattack and was operating normally after the incident.

### 2.1 Recovery strategies

Compromised workstations in the department were completely expunged by hospital IT. The department prioritized the restoration of the Mosaiq R&V to take place first, in order to maintain treatment continuity for roughly 20 patients. After 2 days, the R&V system was restored from a backup made on the night prior to the cyberattack. The clinical team spent many hours checking patient charts and ensuring that dose tracking was consistent and up to date for all patients. Physics conducted extensive validation tests prior to clinical release.

Most systems within radiation oncology were restored in the following weeks. Unfortunately, IT staffing shortages led to a long delay in restoring the Eclipse server. Initially, it was unclear how long the process would take, so a secondary effort began in earnest. The medical physicist had made a recent offline backup of TPS beam data and pre‐calculated and approved beam models, which enabled Varian to establish a temporary TPS, while waiting for the original planning system and database to be (eventually) restored. This temporary TPS required several days of concerted commissioning effort before it was used clinically for a period of 7 days. Serendipitously, this site's beam data were matched to the Eclipse planning system of a nearby affiliated hospital which was not impacted by Institution B's cyberattack. This allowed the site's physicist to compare TPS modeling and planning data to a reference machine, and perform a suite of test calculations with additional assistance from two physicists from the affiliated hospital.

### 2.2 Patient impact

After the local Mosaiq server was restored by mid‐day on day 2, and all validation tests were performed, treatments resumed without incident for the 20‐patient cohort. A smaller cohort (<10) patients experienced 2‐week delays initiating their treatment due to the time spent rebuilding and recommissioning a temporary TPS. After the original Eclipse server was finally restored and validated by the physicists, the clinical plans/dose generated in the temporary TPS were revalidated in the restored TPS.

## 3 INSTITUTION C

This hospital network comprises a central academic tertiary care center and numerous smaller hospitals and affiliates. The attack on October 28, 2020 followed a string of coordinated attacks on several hospitals throughout the United States. This attack, however, resulted from an employee opening a legitimate personal email from a legitimate organization that had, unfortunately, been compromised. Malware was released eventually encrypting more than 1300 servers; contact information of the attackers was provided in a text file, but no ransom was demanded. This attack was likely not targeting the institution specifically. The IT infrastructure team reacted quickly to secure the main site through an immediate shut down of all network access including any access to the internet and patient medical records. As a result, the electronic medical record (EMR), OIS, picture archiving and communication system (PACS), phone, Internet, and email were inaccessible. Physicians had no access to pathology, radiology, lab results, pharmacy, etc. All information located on department shared drives including QA equipment databases, checklists, policies and procedures, and so on could not be accessed. These efforts protected other hospitals in the network but crippled almost all services at the main site. Many systems were down weeks or months while the network was swept, over 5000 computers were reimaged, and servers were rebuilt or declared clean. The network‐wide EMR was down for almost a month.

On the day of the attack, services in radiation oncology were immediately suspended as the OIS was taken offline. An immediate concern was that schedule and patient contact information were not accessible and the hospital phone and emails systems were down making it difficult to contact patients and even know which patients to contact.

### 3.1 Recovery strategies

Fortunately, some systems were not impacted by the attack. For example, the TPS was built on a Unix operating system, and it was still fully operational. Over the following days, weeks, and months, the situation was dynamic and systems were gradually brought back online.

Some immediate strategies were considered to resume treatment, including treatment outside the R&V using stored beams, transfer of patients to other hospitals within the network, and working with the vendor to install a new local Mosaiq server and manually repopulate machine and patient information from the TPS. Transferring patients and treating outside the R&V could be done quickly while rebuilding Mosaiq would take days to weeks. All of these options came with significant challenges and safety concerns.

As there was a complete lack of information about restoration of services, patients were prioritized based on clinical need.^3^ Urgent cases were transferred to a network affiliate while other less urgent patients with complex plans sustained longer delays. Using these strategies, high priority patients had delays of only 2–4 days while it was approaching 2 weeks before prostate and other less urgent patients resumed treatment.

Treating outside the R&V using prebuilt and stored beams was the only path with a definite timeline to resume treatments at the site of attack. A paper charting procedure was put in place involving physicist presence and callout verification for all treatments. Direct transfer of DICOM information from the TPS was not possible. As a result, this use was limited to rectangular fields and electron applicators that could be manually entered and easily verified. The physics team quickly developed in‐house code to parse and transform DICOM data from TPS for treatment outside the R&V, which would allow both static and dynamic treatments to resume. However, for safety concerns it was decided to reserve this technique as a last resort, and it was never used.

To restore full treatment capability, a new Mosaiq server with modified architecture was built. This required significant resources from the IT team and cooperation from the vendor. All plans were reimported and all QA and charting repeated. From time of the attack to time of having a working R&V was 9 days for the first linac and 22 days for all linacs. The original R&V/OIS was eventually restored and manually populated with the downtime data after approximately 3 months.

### 3.2 Patient impact

This cyberattack had consequences for patients. As mentioned above, all patients experienced at least some delay in treatment. Data loss at the time of the attack also resulted in delivery of an incorrect number of fractions (± 1 fraction) for three patients, which was determined after restoration of the legacy OIS. Cancer patients were subject to increased uncertainty and inconveniences as a result of the attack. Staff already under the stress of a pandemic were further burdened by long hours and uncertainty about a path forward. While the entire department came together and put patients first, there were unavoidable limitations to this treatment environment that impacted patient safety.

## 4 SUMMARY AND RECOMMENDATION

The authors wish to convey some key takeaway points to the readers:

1. Cyberattacks are an active threat to the field of radiation oncology.
2. Medical physicists must play a key role in developing contingency plans.
3. Simulated loss of systems scenarios could be performed annually to test readiness.
4. Roles and responsibilities should be defined for crisis management teams.
5. Departmental software systems should be ranked in order of priority so that their restoration can effectively be performed with limited staffing.
6. Access to paper records such as fractionation and contact information could be key in resuming treatments without a legacy information system.
7. Offline backups are crucial for mission‐critical department data; redundancy and failover capability have become commonplace, but these will likely be unavailable in network wide attacks.

In the aftermath of these recent cyberattacks, our institutions instituted policies and procedures to improve our future response to similar events, such as (1) system restoration from clean backups, (2) alternative methods of patient plan transfer, or (3) full system rebuilds. It is also feasible that a set of essential paper records including patient fraction and contact information could be kept and maintained as part of a secure (physical) file storage system. Finally, the most useful contingency plans^4^ will be multifaceted and include foundational changes to how our computer systems are setup from the very beginning. These changes will require collaboration between Medical Physics and IT as well as the vendor. They will be costly both in financial and human capital and will be most easily implemented by large institutions. These vulnerabilities should be considered a significant risk to our business models, and thus vendors also have a vested interest in designing not only more secure systems but systems that can operate safely while other systems such as the R&V are offline. Vendor engagement is paramount in moving to a model that better protects our patients and institutions from cyberattacks.
